# Supplementary material for: Resistance to pyrethroids in Aedes aegypti: Insights into transcriptomic response to different insecticide concentrations
Source: PLoS Negl Trop Dis. 2026 Sep 24;20(9):e0014144. doi: 10.1371/journal.pntd.0014144 (PMC13652323; doi:10.1371/journal.pntd.0014144)
Supplement: Supplementary file 1 [file pntd.0014144.s005.docx]

July 11, 2026

Dr

**Mariangela Bonizzoni**

Academic Editor

PLOS Neglected Tropical Diseases

Re: Response to manuscript PNTD-D_26-00494

We sincerely thank the editors and reviewers for their careful evaluation of our manuscript and for their constructive and insightful comments. We have revised the manuscript accordingly and provided point-by-point responses below. All changes are highlighted in the revised manuscript with Track Changes and red color. We believe the manuscript has been substantially improved and hope it now meets the publication standards of PLOS Neglected Tropical Diseases.

Sincerely,

Omar Triana-Chávez

Corresponding author

**Journal Requirements**

All the requirements were met in the new version.

**Reviewers' comments:**

**Reviewer # 1**

We thank Reviewer #1 for the thorough and constructive evaluation of our manuscript.

1) The objectives and hypothesis are clearly articulated, and the methodology directly addresses the study questions with appropriate analyses and adequate sample sizes.

   **Requests for clarification:** The introduction would benefit from additional programmatic or academic background explaining the rationale for selecting permethrin and lambda-cyhalothrin specifically for this investigation.

**Response:**

We agree and have added a paragraph to the Introduction that explicitly justifies the selection of permethrin and lambda-cyhalothrin. In brief: both insecticides represent the two chemically distinct pyrethroid subclasses (Type I and Type II, distinguished by the presence or absence of an α-cyano group) in wide use for vector control in Colombia and Latin America. Both have been documented in field populations of Ae. aegypti in Colombia, providing direct public health relevance [14,15]. Their contrasting pharmacological effects on the voltage-gated sodium channel also make them ideal for comparative mechanistic studies.

2) Was false discovery rate correction applied to the TopGO testing using Fisher's exact test? If so, please specify the method; if not, please apply FDR correction and revise results accordingly.

**Response:**

Thanks to the reviewer for this inquiry. The algorithms implemented in TopGO (classic, weight01, elim, and parentchild) incorporate an intrinsic correction for the dependency structure inherent in GO hierarchies, in which enrichment of a specific term necessarily implies enrichment of its parent terms. This positive correlation among GO terms violates the assumption of independence required for standard FDR correction methods; applying FDR in this context would therefore be overly stringent and would discard terms with genuine biological relevance. For this reason, we retained the Fisher’s exact test p-value <0.05 threshold without FDR correction, consistent with the recommended practice for TopGO analyses. We specified the use of the TopGO algorithms in the methods section “Annotation with custom database and Gene Ontology (GO)”.

3) What accounts for the inclusion of only two pools for HPR group? Please clarify whether this limitation reflects the number of survivors available following the bioassay and discuss implications for the robustness of conclusions from this group.

**Response:**

The reviewer is correct. We have clarified in the Results section that only two biological replicates were available for the HPR group because one of the three replicates showed inadequate RNA quality during sample processing and was excluded. We have explicitly acknowledged this as a limitation, noting that conclusions drawn exclusively from the HPR group should be interpreted with appropriate caution given the reduced statistical power. Wherever possible, HPR-based observations are supported by data from the other groups. Importantly, DESeq2 addresses low replicate numbers through its size factor normalization and the estimateDispersion function. As stated by Love et. al. in the original DESeq2 publication [2], using the mean expression across all genes in the dataset, “two or three replicates are common and reasonable”. Furthermore, DEGs were not selected through pairwise comparison alone; instead, we applied an incremental stepwise expression pattern approach (HXR > HXS > LX) across three phenotypically separated groups, which substantially reduces the probability of false positives arising from the limited replication in the HPR group. This limitation is acknowledged in the final paragraph, after the Discussion section (“Limitations”).

4) The baseline resistance present in the AF strain warrants more discussion. Although kdr mutations will naturally be contributing in part to this phenotype, how might this pre-existing resistance phenotype influence the differential gene expression patterns observed in the RNAseq results?

**Response:**

This is an important point. We have added a paragraph in the Discussion (kdr section) addressing how the pre-existing resistance background of the AF strain — particularly the near-fixation of the F1534C allele — may influence the transcriptomic profiles observed. Briefly, constitutive target-site resistance may reduce selective pressure on metabolic genes in the AF background, and the use of AF as the reference baseline in DEG comparisons means that any constitutively elevated gene expression shared between AF and the selected strains would not be captured. We discuss how this should be considered when interpreting the magnitude of differential expression.

5) Have you considered supplementing the current analysis with RNAseq data screening for SNPs within metabolic resistance candidate genes (e.g. CYP304B2, CYP4H32 etc.) to identify those increasing in frequency across the HXR > HXS > LX hierarchy? This could provide mechanistic insight into the genetic basis of resistance gradation. SNP calling using these RNAseq data could be validated according to kdr mutation frequency identified using AS-PCR.

**Response:**

We thank the reviewer for this suggestion, which would add valuable mechanistic depth. While this analysis is beyond the scope of the current study — and the RNAseq data were generated primarily for expression profiling rather than variant calling — we acknowledge its merit and have added this as a specific direction for future research in the Discussion section. We note that our kdr data (AS-PCR) could serve as a validation framework for any future SNP calling approach, as the reviewer suggests.

6) A formal definition of the "cuticle" gene category is required. Please specify whether this classification is based on BLAST annotation alone, and explicitly state which genes are included (e.g., only structural cuticular proteins and chitin metabolism genes, or also fatty acid metabolism-related enzymes?). Provide a supplementary annotation scheme for clarity.

**Response:**

We have added a formal definition of "cuticle genes" in the Results section (permethrin subsection), specifying that this category includes structural cuticular proteins (CPR, CPAP, and analogous families) and enzymes directly involved in chitin metabolism (chitin synthases, chitinases, chitin deacetylases), classified based on BLAST annotation against UniProt/SwissProt and VectorBase gene descriptions. A complete annotated list is provided in the supplementary tables (Supplementary Table S3).

7) Why were UDP-glucuronosyltransferases (UGTs) not investigated alongside the other canonical metabolic resistance genes examined (CYP450s, GSTs, ABCs, CCE)? If this was due to there being none in the filtered gene list then please specify, otherwise recommended to highlight UGTs alongside the other metabolic IR gene families

**Response:**

We thank the reviewer for this comment. No UGT genes met the expression threshold criteria applied in our filtered DEG list. We have explicitly stated this in the manuscript (Results, permethrin section): "We also searched for UDP-glucuronosyltransferases (UGTs) within the filtered DEG list; however, no UGT genes met the expression threshold criteria applied in this study, and they are therefore not discussed further." This clarification prevents the reader from assuming UGTs were overlooked. However, we highlighted this gene category in the volcano plot and added it to the supplementary Table 3 as “UGT” due to its relevance to insecticide resistance that could be explored further. This and other supporting information for the inclusion of specific gene terms in the analysis were added in lines 294 to 309 in the method section, “Annotation with custom database and Gene Ontology (GO)”.

8) The examination of the alcohol dehydrogenase (ADH) family in insecticide-resistant DEG analysis appears without sufficient precedent. Whilst I agree that these genes are likely involved in insecticide metabolism, please provide more rationale for their inclusion in the analysis.

**Response:**

We thank the reviewer for this observation. One of our interests was in identifying overlooked mechanisms of insecticide resistance. In this sense, we searched for ADH, which belongs to short-chain dehydrogenases with a plethora of functions, including xenobiotic metabolism [4]. Moreover, in other models, ADH has been found to interact with a CYP6 promoter regulating its expression, supporting our search for this category [5]. For these reasons, we added this annotation to the analysis. This and other supporting information for the inclusion of specific gene terms in the analysis were added in the method section “Annotation with custom database and Gene Ontology (GO)”.

9) The manuscript mentions "some interesting genes that did not surpass the stringent threshold," including CLIP domain serine proteases and caspases. The relevance of these genes requires clarification, considering they did not pass the filters described. Whilst the mechanistic roles these genes may play in the context of insecticide resistance is considered in the discussion section, the narrative flow in the results section is rather abrupt. Please add a line along the lines of "genes involved in oxidative stress, cell death, and immunity" etc., so the reader is aware why these genes are highlighted in the results section.

**Response:**

We have added a clarifying sentence after the mention of these genes, stating that they are highlighted because they are involved in oxidative stress responses, innate immune signaling, and apoptotic cell death pathways — processes relevant to insecticide-induced cellular damage and survival. This bridges the Results and Discussion sections and gives the reader immediate context for their inclusion.

10) While I agree that the inclusion of analysis on odorant-binding proteins (OBPs) is certainly relevant and should be examined further in IR phenotypes, the rationale for examining these genes in the permethrin-exposed group but not the lambda-cyhalothrin-exposed group requires explicit explanation. Presumably, OBPs comprised a large proportion of the upregulated IR DEG list in the permethrin group only, but this should be clearly stated in the text.

**Response:**

We thank the reviewer for this comment. We have added an explanatory sentence in the Results (permethrin subsection): OBP genes were analyzed specifically in the permethrin-exposed group because they comprised a disproportionately large fraction (>30%) of the upregulated IR DEGs in the HPR group — a pattern that was not observed in the lambda-cyhalothrin-exposed populations, where OBPs did not emerge among the top-regulated transcripts. This differential enrichment justified the focused OBP analysis in the permethrin context. Additionally, It is plausible that OBPs function as molecular buffers for xenobiotics, and the over-expression of these transcripts in tissues with abundant cuticle could diminish the insecticide passage through hemolymph [8].

11) The upregulation of energy-related genes following Type I pyrethroid exposure has been documented in other mosquito studies, and may not be specific to Type II as noted in this study. The current discussion should integrate the broader literature to contextualise whether this pattern is specific to the conditions examined or represents a more general physiological response to this insecticide class.

**Response:**

We have integrated a paragraph in the Discussion that contextualizes our findings within the broader literature. We note that upregulation of energy metabolism and mitochondrial function genes following pyrethroid exposure has been documented in Culex quinquefasciatus and Anopheles gambiae [56,57], suggesting a broadly conserved physiological response. We also discuss why our results — predominantly associating this pattern with lambda-cyhalothrin (Type II) rather than permethrin (Type I) — suggest that while energy metabolism responses may be general, their direction and magnitude differ between pyrethroid types and genetic backgrounds.

12)  Please provide a supplementary file containing the complete differential expression results for all analysed groups. Each gene entry should be annotated according to the functional categories described in the Results (CYP450, cuticular proteins, ADH, UGTs, etc.). This will greatly facilitate comparison for future studies and improve the transparency and utility of the data.

**Response:**

We agree with the reviewer. We will prepare and upload as Supporting Information a comprehensive supplementary table containing the full DEG results for all groups, with each gene annotated according to the functional categories described in the Results (CYP450, cuticular proteins, ADH, UGTs, etc.). This will be uploaded prior to resubmission. We include a new column, “Category”, in Supplementary Table 3 that contains all the genes and their respective IR terms.

13) Consider using Figure 9 as the graphical abstract, as it effectively summarises the key findings.

**Response:**

We appreciate this suggestion. We will upload Figure 9 as the graphical abstract upon resubmission, as it provides a clear visual summary of the transcriptomic differences between lambda-cyhalothrin and permethrin responses.

Reviewer #2: It is an interesting study. However, I have two concerns that require further clarification:

We thank Reviewer #2 for identifying two important points that required further clarification.

1. Why were LC25 and LC75 used for the selection pressure, rather than LC50, which is more commonly used in resistance selection studies? Please justify the rationale for choosing these concentrations.

**Response:**

The choice of LC25 and LC75 was deliberate and reflects our experimental objective to model two ecologically distinct insecticide exposure scenarios. LC25 approximates the sublethal concentrations frequently encountered in the field due to environmental degradation, dilution, incomplete spray coverage, or behaviors that reduce mosquito exposure (e.g., resting avoidance) [8,9]. LC75, conversely, simulates the high insecticidal pressure achieved under optimal spraying conditions or indoor residual applications. By using this biologically and operationally relevant concentration gradient rather than a single LC50, we were able to characterize resistance mechanisms across a range of conditions applicable to real-world vector control programs. We have added this justification explicitly in the Discussion section of the revised manuscript.

2. It is unusual to observe such a significant increase in resistance within just one generation, particularly up to 12.3-fold. The authors should provide further explanation and discussion for this rapid change.

**Response:**

We thank the reviewer for raising this important point. The 12.3-fold increase in resistance ratio (RR50) observed in the HL strain after two generations of selection at LC75 is striking, but we believe it is explained by the genetic background of the starting population. The AF strain, originally collected from Acacías, Meta (a region with documented history of pyrethroid use [14]), already harbored elevated kdr allele frequencies and a pre-existing resistance phenotype. High-pressure selection (LC75) acts as a strong sieve that rapidly enriches for resistant genotypes already present at elevated frequencies in this field-derived population, rather than requiring the emergence of novel resistance mutations de novo. This mechanism — enrichment of standing genetic variation — can produce rapid and large-magnitude resistance increases under intense selection, as has been reported in other Ae. aegypti populations selected in laboratory settings [51]. We note that two generations of selection are applied here, not one, and the cumulative effect of two cycles of high-pressure selection on a pre-resistant starting population is consistent with this magnitude of change. This explanation has been incorporated into the Discussion section.

 We hope that these revisions adequately address all concerns raised. We believe the manuscript is substantially improved and look forward to your further consideration.

Sincerely,

**Omar Triana-Chávez**

Corresponding Author

E-mail: [omar.triana@udea.edu.co](mailto:omar.triana@udea.edu.co)
