# Supplementary material for: Resistance to pyrethroids in Aedes aegypti: Insights into transcriptomic response to different insecticide concentrations
Source: PLoS Negl Trop Dis. 2026 Sep 24;20(9):e0014144. doi: 10.1371/journal.pntd.0014144 (PMC13652323; doi:10.1371/journal.pntd.0014144)
Supplement: Peer review history

# Peer review: Decision Letter 0

**DOI:** 10.1371/journal.pntd.0014144.r001

**Authors:** Nigel Beebe, Mariangela Bonizzoni

13 May 2026

Response to Reviewers Revised Manuscript with Track Changes Manuscript

Kind regards,

Shaden Kamhawi

co-Editor-in-Chief

PLOS Neglected Tropical Diseases

orcid.org/0000-0003-4304-636XX

Paul Brindley

co-Editor-in-Chief

PLOS Neglected Tropical Diseases

orcid.org/0000-0003-1765-0002

**Journal Requirements:**

Please respond directly to this email and provide any known details concerning your material's license terms and permissions required for reuse, even if you have not yet obtained copyright permissions or are unsure of your material's copyright compatibility. Once you have responded and addressed all other outstanding technical requirements, you may resubmit your manuscript within Editorial Manager.

Potential Copyright Issues

**Reviewers' comments:**

**Key Review Criteria Required for Acceptance?**

As you describe the new analyses required for acceptance, please consider the following:

**Methods**

-Are the objectives of the study clearly articulated with a clear testable hypothesis stated?

-Is the study design appropriate to address the stated objectives?

-Is the population clearly described and appropriate for the hypothesis being tested?

-Is the sample size sufficient to ensure adequate power to address the hypothesis being tested?

-Were correct statistical analysis used to support conclusions?

-Are there concerns about ethical or regulatory requirements being met?

Reviewer #1: The objectives and hypothesis are clearly articulated, and the methodology directly addresses the study questions with appropriate analyses and adequate sample sizes.

Requests for clarification:

The introduction would benefit from additional programmatic or academic background explaining the rationale for selecting permethrin and lambda-cyhalothrin specifically for this investigation.

Was false discovery rate correction applied to the TopGO testing using Fisher's exact test? If so, please specify the method; if not, please apply FDR correction and revise results accordingly.

What accounts for the inclusion of only two pools for HPR group? Please clarify whether this limitation reflects the number of survivors available following the bioassay, and discuss implications for the robustness of conclusions from this group.

The baseline resistance present in the AF strain warrants more discussion. Although kdr mutations will naturally be contributing in part to this phenotype, how might this pre-existing resistance phenotype influence the differential gene expression patterns observed in the RNAseq results?

Have you considered supplementing the current analysis with RNAseq data screening for SNPs within metabolic resistance candidate genes (e.g. CYP304B2, CYP4H32 etc.) to identify those increasing in frequency across the HXR > HXS > LX hierarchy? This could provide mechanistic insight into the genetic basis of resistance gradation. SNP calling using these RNAseq data could be validated according to kdr mutation frequency identified using AS-PCR.

Reviewer #2: see below

**********

**Results**

-Does the analysis presented match the analysis plan?

-Are the results clearly and completely presented?

-Are the figures (Tables, Images) of sufficient quality for clarity?

Reviewer #1: The results are generally well explained and clearly presented. The systemic narrative framework effectively contextualises shifts in gene expression patterns across each group. The clear separation between Type I and Type II pyrethroid patterns is particularly well described. All figures are of high quality and suitable for publication.

Requests for clarification:

A formal definition of the "cuticle" gene category is required. Please specify whether this classification is based on BLAST annotation alone, and explicitly state which genes are included (e.g., only structural cuticular proteins and chitin metabolism genes, or also fatty acid metabolism-related enzymes?). Provide a supplementary annotation scheme for clarity.

Why were UDP-glucuronosyltransferases (UGTs) not investigated alongside the other canonical metabolic resistance genes examined (CYP450s, GSTs, ABCs, CCE)? If this was due to there being none in the filtered gene list then please specify, otherwise recommended to highlight UGTs alongside the other metabolic IR gene families.

The examination of the alcohol dehydrogenase (ADH) family in insecticide-resistant DEG analysis appears without sufficient precedent. Whilst I agree that these genes are likely involved in insecticide metabolism, please provide more rationale for their inclusion in the analysis.

The manuscript mentions "some interesting genes that did not surpass the stringent threshold," including CLIP domain serine proteases and caspases. The relevance of these genes requires clarification, considering they did not pass the filters described. Whilst the mechanistic roles these genes may play in the context of insecticide resistance is considered in the discussion section, the narrative flow in the results section is rather abrupt. Please add a line along the lines of "genes involved in oxidative stress, cell death, and immunity" etc., so the reader is aware why these genes are highlighted in the results section.

While I agree that the inclusion of analysis on odorant-binding proteins (OBPs) is certainly relevant and should be examined further in IR phenotypes, the rationale for examining these genes in the permethrin-exposed group but not the lambda-cyhalothrin-exposed group requires explicit explanation. Presumably, OBPs comprised a large proportion of the upregulated IR DEG list in the permethrin group only, but this should be clearly stated in the text.

The upregulation of energy-related genes following Type I pyrethroid exposure has been documented in other mosquito studies, and may not be specific to Type II as noted in this study. The current discussion should integrate the broader literature to contextualise whether this pattern is specific to the conditions examined or represents a more general physiological response to this insecticide class.

Reviewer #2: see below

**********

**Conclusions**

-Are the conclusions supported by the data presented?

-Are the limitations of analysis clearly described?

-Do the authors discuss how these data can be helpful to advance our understanding of the topic under study?

-Is public health relevance addressed?

Reviewer #1: The conclusions are well supported by the data presented. The study places results in a clear public health context, and the relevance of findings to insecticidal tools for vector control is appropriately addressed.

Reviewer #2: see below

**********

**Editorial and Data Presentation Modifications?**

Use this section for editorial suggestions as well as relatively minor modifications of existing data that would enhance clarity. If the only modifications needed are minor and/or editorial, you may wish to recommend “Minor Revision” or “Accept”.

Reviewer #1: Please provide a supplementary file containing the complete differential expression results for all analysed groups. Each gene entry should be annotated according to the functional categories described in the Results (CYP450, cuticular proteins, ADH, UGTs, etc.). This will greatly facilitate comparison for future studies and improve the transparency and utility of the data.

Consider using Figure 9 as the graphical abstract, as it effectively summarises the key findings.

Reviewer #2: see below

**********

**Summary and General Comments**

Use this section to provide overall comments, discuss strengths/weaknesses of the study, novelty, significance, general execution and scholarship. You may also include additional comments for the author, including concerns about dual publication, research ethics, or publication ethics. If requesting major revision, please articulate the new experiments that are needed.

Reviewer #1: This is a skilfully designed study and presents a well-executed investigation of pyrethroid resistance mechanisms in mosquitoes, with several notable strengths:

The systemic narrative framework effectively integrates gene expression patterns across treatment groups, providing coherent biological interpretation.

The integrated assessment of kdr genotyping, metabolism assays, and RNAseq results is sophisticated and strengthens mechanistic conclusions.

Type I and Type II pyrethroid-induced expression patterns are clearly differentiated and well described.

The speculation regarding microbiota-SLIMP interactions is novel and intriguing.

The discussion demonstrates critical thinking and balanced representation of findings, with appropriate acknowledgment of limitations.

The main limitation of the current manuscript is clarification why some genes/gene families were highlighted in the text, and how these genes were grouped into corresponding gene families.

Reviewer #2: It is an interesting study. However, I have two concerns that require further clarification:

1. Why were LC25 and LC75 used for the selection pressure, rather than LC50, which is more commonly used in resistance selection studies? Please justify the rationale for choosing these concentrations.

2. It is unusual to observe such a significant increase in resistance within just one generation, particularly up to 12.3-fold. The authors should provide further explanation and discussion for this rapid change.

**********

PLOS authors have the option to publish the peer review history of their article (what does this mean?). If published, this will include your full peer review and any attached files.

If you choose “no”, your identity will remain anonymous but your review may still be made public.

**Do you want your identity to be public for this peer review?**  For information about this choice, including consent withdrawal, please see our Privacy Policy.

Reviewer #1: **Yes:**  Henry A. Youd

Reviewer #2: No

**Figure resubmission:**

**Reproducibility:**

To enhance the reproducibility of your results, we recommend that authors of applicable studies deposit laboratory protocols in protocols.io, where a protocol can be assigned its own identifier (DOI) such that it can be cited independently in the future. Additionally, PLOS ONE offers an option to publish peer-reviewed clinical study protocols. Read more information on sharing protocols at https://plos.org/protocols?utm_medium=editorial-email&utm_source=authorletters&utm_campaign=protocols

# Author comment: Author response to Decision Letter 1

**DOI:** 10.1371/journal.pntd.0014144.r002

28 Jul 2026

Attachment Submitted filename: Response_to_reviewers.docx

# Peer review: Decision Letter 1

**DOI:** 10.1371/journal.pntd.0014144.r003

**Authors:** Nigel Beebe

15 Sep 2026

Dear Dr Triana-Chávez,

We are pleased to inform you that your manuscript 'Resistance to Pyrethroids in Aedes aegypti: Insights into Transcriptomic Response to Different Insecticide Concentrations' has been provisionally accepted for publication in PLOS Neglected Tropical Diseases.

Before your manuscript can be formally accepted you will need to complete some formatting changes, which you will receive in a follow up email. A member of our team will be in touch with a set of requests.

Please note that your manuscript will not be scheduled for publication until you have made the required changes, so a swift response is appreciated.

IMPORTANT: The editorial review process is now complete. PLOS will only permit corrections to spelling, formatting or significant scientific errors from this point onwards. Requests for major changes, or any which affect the scientific understanding of your work, will cause delays to the publication date of your manuscript.

Should you, your institution's press office or the journal office choose to press release your paper, you will automatically be opted out of early publication. We ask that you notify us now if you or your institution is planning to press release the article. All press must be co-ordinated with PLOS.

Thank you again for supporting Open Access publishing; we are looking forward to publishing your work in PLOS Neglected Tropical Diseases.

Best regards,

Nigel Beebe, PhD

Section Editor

PLOS Neglected Tropical Diseases

Nigel Beebe

Section Editor

PLOS Neglected Tropical Diseases

Shaden Kamhawi

co-Editor-in-Chief

PLOS Neglected Tropical Diseases

orcid.org/0000-0003-4304-636XX

Paul Brindley

co-Editor-in-Chief

PLOS Neglected Tropical Diseases

orcid.org/0000-0003-1765-0002

***********************************************************

p.p1 {margin: 0.0px 0.0px 0.0px 0.0px; line-height: 16.0px; font: 14.0px Arial; color: #323333; -webkit-text-stroke: #323333}span.s1 {font-kerning: none

Reviewer's Responses to Questions

**Key Review Criteria Required for Acceptance?**

As you describe the new analyses required for acceptance, please consider the following:

**Methods**

-Are the objectives of the study clearly articulated with a clear testable hypothesis stated?

-Is the study design appropriate to address the stated objectives?

-Is the population clearly described and appropriate for the hypothesis being tested?

-Is the sample size sufficient to ensure adequate power to address the hypothesis being tested?

-Were correct statistical analysis used to support conclusions?

-Are there concerns about ethical or regulatory requirements being met?

Reviewer #1: The objectives and hypothesis are clearly articulated, and the methodology directly addresses the study questions with appropriate analyses and adequate sample sizes. All revision requests have been adequately addressed.

Reviewer #2: n/a

**********

**Results**

-Does the analysis presented match the analysis plan?

-Are the results clearly and completely presented?

-Are the figures (Tables, Images) of sufficient quality for clarity?

Reviewer #1: The results are generally well explained and clearly presented. The systemic narrative framework effectively contextualises shifts in gene expression patterns across each group. The clear separation between Type I and Type II pyrethroid patterns is particularly well described. All figures are of high quality and suitable for publication, and all necessary supplementary files provided. All revision requests have been adequately addressed.

Reviewer #2: n/a

**********

**Conclusions**

-Are the conclusions supported by the data presented?

-Are the limitations of analysis clearly described?

-Do the authors discuss how these data can be helpful to advance our understanding of the topic under study?

-Is public health relevance addressed?

Reviewer #1: The conclusions are well supported by the data presented. The study places results in a clear public health context, and the relevance of findings to insecticidal tools for vector control is appropriately addressed.

Reviewer #2: n/a

**********

**Editorial and Data Presentation Modifications?**

Use this section for editorial suggestions as well as relatively minor modifications of existing data that would enhance clarity. If the only modifications needed are minor and/or editorial, you may wish to recommend “Minor Revision” or “Accept”.

Reviewer #1: (No Response)

Reviewer #2: n/a

**********

**Summary and General Comments**

Use this section to provide overall comments, discuss strengths/weaknesses of the study, novelty, significance, general execution and scholarship. You may also include additional comments for the author, including concerns about dual publication, research ethics, or publication ethics. If requesting major revision, please articulate the new experiments that are needed.

Reviewer #1: As previously outlined, this is a skilfully designed study and presents a well-executed investigation of pyrethroid resistance mechanisms in mosquitoes, with several notable strengths:

The systemic narrative framework effectively integrates gene expression patterns across treatment groups, providing coherent biological interpretation.

The integrated assessment of kdr genotyping, metabolism assays, and RNAseq results is sophisticated and strengthens mechanistic conclusions.

Type I and Type II pyrethroid-induced expression patterns are clearly differentiated and well described.

The speculation regarding microbiota-SLIMP interactions is novel and intriguing.

The discussion demonstrates critical thinking and balanced representation of findings, with appropriate acknowledgment of limitations.

Following the revisions from the authours, the manuscript text now has greater readability, motivations for particular methodological choices are clearer, and justification for highlighting of certain genes and gene families has been improved.

Reviewer #2: Revision is satisfactory.

**********

PLOS authors have the option to publish the peer review history of their article (what does this mean?). If published, this will include your full peer review and any attached files.

If you choose “no”, your identity will remain anonymous but your review may still be made public.

**Do you want your identity to be public for this peer review?**  For information about this choice, including consent withdrawal, please see our Privacy Policy.

Reviewer #1: **Yes:**  Henry A. Youd

Reviewer #2: No

# Editor report: Acceptance letter

**DOI:** 10.1371/journal.pntd.0014144.r004

**Authors:** Nigel Beebe

Dear Dr Triana-Chávez,

We are delighted to inform you that your manuscript, "Resistance to Pyrethroids in Aedes aegypti: Insights into Transcriptomic Response to Different Insecticide Concentrations," has been formally accepted for publication in PLOS Neglected Tropical Diseases.

We have now passed your article onto the PLOS Production Department who will complete the rest of the publication process. All authors will receive a confirmation email upon publication.

The corresponding author will soon be receiving a typeset proof for review, to ensure errors have not been introduced during production. Please review the PDF proof of your manuscript carefully, as this is the last chance to correct any scientific or type-setting errors. Please note that major changes, or those which affect the scientific understanding of the work, will likely cause delays to the publication date of your manuscript. Note: Proofs for Front Matter articles (Editorial, Viewpoint, Symposium, Review, etc...) are generated on a different schedule and may not be made available as quickly.

Soon after your final files are uploaded, the early version of your manuscript will be published online unless you opted out of this process. The date of the early version will be your article's publication date. The final article will be published to the same URL, and all versions of the paper will be accessible to readers.

For Research Articles, you will receive an invoice from PLOS for your publication fee after your manuscript has reached the completed accept phase. If you receive an email requesting payment before acceptance or for any other service, this may be a phishing scheme. Learn how to identify phishing emails and protect your accounts at https://explore.plos.org/phishing.

Thank you again for supporting open-access publishing; we are looking forward to publishing your work in PLOS Neglected Tropical Diseases.

Best regards,

Shaden Kamhawi

co-Editor-in-Chief

PLOS Neglected Tropical Diseases

Paul Brindley

co-Editor-in-Chief

PLOS Neglected Tropical Diseases
